# Durable Global Correction of CNS and PNS and Lifespan Rescue in Murine Globoid Cell Leukodystrophy via AAV9-Mediated Monotherapy

**DOI:** 10.3390/cells14241942

**Published:** 2025-12-08

**Authors:** Dar-Shong Lin, Che-Sheng Ho, Yu-Wen Huang, Tsung-Han Lee, Zo-Darr Huang, Tuan-Jen Wang, Wern-Cherng Cheng, Sung-Fu Huang

**Affiliations:** 1 Department of Translational Medicine, MacKay Memorial Hospital, Taipei 104217, Taiwan; 2 Department of Medicine, MacKay Medical University, New Taipei 252005, Taiwan; 3 Department of Pediatrics, MacKay Memorial Hospital, Taipei 104217, Taiwan; 4 Department of Neurology, MacKay Children’s Hospital, Taipei 104217, Taiwan; 5 Department of Medical Research, MacKay Memorial Hospital, Taipei 104217, Taiwan; 6 Department of Laboratory Medicine, MacKay Memorial Hospital, Taipei 104217, Taiwan; 7 Department of Laboratory Medicine, School of Medicine, College of Medicine, National Taiwan University, Taipei 106319, Taiwan

**Keywords:** AAV, globoid cell leukodystrophy, GALC, gene therapy, psychosine, demyelination, neuroinflammation, autophagy

## Abstract

**Highlights:**

**What are the main findings?**

- A single, region-specific intracranial dose of AAV9-GALC achieves widespread CNS-PNS biodistribution, sustained supraphysiological GALC activity, and complete lifelong psychosine normalization in Twitcher mice.
- This streamlined monotherapy preserves myelin integrity, proteostasis, and motor function, leading to near–wild-type lifespan without the need for HSCT, repeated dosing, multi-route administration, or high systemic AAV exposure.

**What are the implications of the main findings?**

- Demonstrating durable metabolic and structural correction after a single intracranial dose provides strong rationale for translational intracerebral AAV9 strategies that minimize procedural burden and systemic risks.
- These results establish a practical therapeutic framework for GLD, highlighting the feasibility of achieving long-term CNS-PNS wide correction through targeted, low-exposure gene delivery.

**Abstract:**

Globoid cell leukodystrophy (GLD) is a devastating lysosomal storage disorder caused by galactocerebrosidase (GALC) deficiency, leading to cytotoxic psychosine accumulation, broad neuroinflammation, dysfunction of autophagy and ubiquitin-proteasome system, progressive demyelination in both the central (CNS) and peripheral nervous systems (PNS), and premature death. Curative treatments are lacking, highlighting the urgent need for transformative approaches. Existing therapies have failed to achieve durable metabolic correction across neural compartments or sustained functional recovery. Here, we demonstrate that a single intracranial administration of high-titer AAV9-GALC targeting the thalamus and deep cerebellar nuclei achieves unprecedented and lifelong therapeutic efficacy in the Twitcher mouse model of GLD. This region-specific monotherapy achieved broad neuronal and glial transduction throughout the CNS and PNS, resulting in sustained supraphysiological GALC activity and complete normalization of psychosine levels. Treated mice exhibited preserved proteostasis, axonal architecture, and myelin integrity, inhibition of neuroinflammation, alongside restored motor function. Remarkably, treated mice attain lifespans approaching wild-type levels, far surpassing all previously reported interventions in this model, indicating a durable, possibly lifelong therapeutic effect. By achieving durable and comprehensive metabolic and structural correction across neural systems without repeated dosing, multi-route delivery, combinational therapy, hematopoietic stem cell transplantation, or high-dose systemic delivery, this study establishes CNS-directed AAV9 monotherapy as a clinically translatable and potentially lifelong therapeutic paradigm for GLD.

## 1. Introduction

Globoid cell leukodystrophy (GLD), or Krabbe disease, is caused by lysosomal storage disease. The infantile form of GLD accounts for approximately 90% of cases and represents the most severe phenotype. Affected infants typically present with extreme irritability, spasticity, and feeding difficulties as early as two months of age [1]. These symptoms rapidly progress to include vision loss, seizures, developmental regression, and profound neurodegeneration, culminating in early mortality, often by the age of two years [1]. The sole established therapeutic intervention for infantile GLD is hematopoietic stem cells transplantation (HSCT) for presymptomatic patients, extending median survival to 15.5 years but failing to halt neurological decline [2].

GLD is caused by autosomal recessive mutations in the GALC gene, which encodes lysosomal galactocerebrosidase, an enzyme essential for galactosylceramide degradation, a key sphingolipid component of myelin [1]. GALC deficiency results in impaired catabolism of both galactosylceramide and galactosylsphingosine (psychosine), the latter being a cytotoxic metabolite that accumulates in the central nervous system (CNS) and peripheral nervous system (PNS). Psychosine integrates into lipid rafts, disrupting membrane architecture and intracellular signaling critical for oligodendrocyte survival [3]. Additionally, psychosine interferes with autophagy and the ubiquitin-proteasome system (UPS), leading to cytoplasmic aggregate formation, mitochondrial dysfunction, increased reactive oxygen species, and apoptotic death of myelin-forming cells [4,5].

The Twitcher (Twi) mouse, an authentic murine model of infantile GLD, harbors a spontaneous nonsense mutation in Galc [6]. These mice exhibit progressive neurological decline that includes tremors, ataxia, paralysis of the hindlimb, reduced food intake, and weight loss, eventually succumbing around postnatal day 40 (P40). Pathologically, Twi mice exhibit absent GALC activity, elevated psychosine levels, widespread demyelination, and astrogliosis in the CNS and PNS, closely recapitulating human disease [7]. This model is extensively used to investigate disease mechanisms and evaluate emerging therapeutic strategies.

Several therapeutic approaches have been explored in Twi mice, including HSCT, enzyme replacement therapy (ERT), substrate reduction therapy (SRT), and gene therapy. HSCT has demonstrated benefits in reducing neuroinflammation and partially delaying disease progression, particularly when administered pre-symptomatically [7]. ERT has demonstrated limited effectiveness due to challenges in achieving sufficient enzyme delivery across the blood-brain barrier [8]. SRT achieves modest reductions in psychosine accumulation, though it has limited impact on survival [9]. Gene therapy using adeno-associated viral (AAV) vectors has shown the most promising approach to date, especially when administered neonatally, significantly extending lifespan and preserving myelin integrity [10]. While no single therapy fully reverses Krabbe disease, combinatorial strategies addressing enzyme deficiency, substrate toxicity, and neuroinflammation show the most promise in pre-clinical studies [10]. Yet, effective interventions restoring long-term central and peripheral myelin integrity, metabolism, and survival remain limited.

In this study, we demonstrate comprehensive correction of pathological, metabolic, and functional deficits in Twitcher mice through a single-dose, CNS-directed AAV9-GALC monotherapy. This targeted approach achieves sustained supraphysiological GALC activity, normalization of psychosine levels across both the CNS and PNS, and long-term preservation of proteostasis and myelin integrity. Notably, this treatment extends lifespan to near wild-type levels, surpassing all previously reported outcomes from either monotherapy or combinatorial gene therapy strategies. By achieving durable correction without repeated administration, combination therapy, HSCT, or high-dose systemic delivery, this study establishes CNS-directed AAV9 monotherapy as a clinically translatable and potentially lifelong therapeutic paradigm for GLD.

## 2. Materials and Methods

### 2.1. Animals and Therapy

All animal procedures were conducted in accordance with the guidelines for animal care sanctioned by the Animal Care and Use Committee of MacKay Memorial Hospital. The colonies of heterozygous (twi/+) twitcher mic, possessing a genetic background of C57BL/6J, were maintained through inbreeding under pathogen-free conditions in the animal research facility at MacKay Memorial Hospital. Genotyping of twitcher mice was performed at three days of age using a molecular PCR assay, as previously described [11]. Experimental subjects, including wild-type (+/+) and homozygous (twi/twi) mice, were allowed to live freely until reaching the moribund stage.

Newborn Twi mice were injected with AAV9-GALC at P3. The viral suspension was slowly injected into thalamus (1.5 mm rostral and 1 mm lateral from the lambda and 3 mm deep), and cerebellum (2 mm caudal and 1.5 mm lateral from the lumbda and 30° medial oblique 3 mm deep) at both hemispheres with Hamilton syringe (Hamilton Company, Reno, NV, USA). The animals received a total of 1.2 × 10^12^ AAV9 particles in a volume of 5 μL and 10 μL at each thalamus and deep cerebellum injection site, respectively. Untreated Twi and normal wild-type (WT) mice were included as controls.

### 2.2. Vector Production

The preparation of recombinant adeno-associated virus (rAAV) vectors was performed using a cotransfection method at the AAV Core Facility of Academia Sinica. The AAV9-GALC vector was designed to incorporate murine GALC cDNA under the regulation of the human cytomegalovirus enhancer and chicken-β-actin promoter. The recombinant AAV9-GALC vector was packed through a cotransfection procedure, involving vector plasmids and helper plasmids. The purification of the viral vector was performed by means of cesium chloride gradient centrifugation followed by extensive dialysis. The AAV titter was determined by droplet digital PCR.

### 2.3. GALC Activity

GALC activity was assessed using a previously described modified method [12]. Fresh tissue samples were homogenized in 10 mM sodium phosphate buffer (pH 6.0) containing 0.1% (*v*/*v*) NP-40, followed by centrifugation. An aliquot of 50 μL supernatant (5 μg protein) was incubated at 37 °C for 1.5 h in 100 μL reaction buffer (0.1/0.2 M citrate/phosphate buffer, pH 4, and 22 mM AgNO_3_) containing 1.5 mM 4-methylumbelliferyl-D-galactopyranoside (cat#38597-12-5, Sigma-Aldrich, St Louis, MO, USA) as fluorescent substrate. The reaction was terminated by adding 200 μL stop buffer (0.2 M glycine/NaOH, pH 10.6). The fluorescence of released 4-methylumbelliferone was measured on a DTX 880 Multimode Detector (Beckman Coulter, Brea, CA, USA) at excitation and emission wavelengths of 385 nm and 450 nm, respectively. Enzymatic activity was expressed as nmol/mg/h.

### 2.4. Psychosine Concentration

Psychosine levels were quantified using a previously described LC-MS method with slight modifications [13,14]. Psychosine (cat#1305) and N-acetyl-psychosine (cat#1325) (internal standard, IS) were obtained from Matreya Chemical Co. (Ann Arbor, MI, USA).

Fresh tissue samples were homogenized in ice cold methanol (20% *w*/*v*). An 80 μL aliquot of homogenate was mixed with 240 μL of a formic acid/ethanol/isopropanol/methanol solution (0.5:37.5:37.5:25, *v*/*v*/*v*/*v*) and 10 μL of 50 ng/mL IS. The mixture was centrifuged at 20,400× *g* for 5 min and 150 μL of the resulting supernatant was combined with 60 μL of 1,3-butanediol. The mixture was evaporated to dryness under a nitrogen stream at 60 °C, reconstituted in 300 μL of mobile phase B (0.1% formic acid in acetonitrile/methanol, 95:5, *v*/*v*), ultrasonicated for 5 min and centrifuged at 1220× *g* for 10 min. The supernatant was filtered through a 0.22 μm membrane filter and analyzed using an Agilent 1260 infinity LC system (Agilent Technologies, Sata Clara, CA, USA). Psychosine levels were expressed as pmole/mg weight.

### 2.5. Immunofluorescences

Immunofluorescence staining was performed on slide mounted mouse brain cryosections according to an optimized protocol. Briefly, tissues were fixed in 4% paraformaldehyde (PFA), sequentially cryoprotected in 30%, 40% and 60% sucrose solutions, embedded in optimal cutting temperature (OCT) compound, and snap-frozen in chilled isopentane.

The sections were allowed to equilibrate to room temperature and post-fixed in 4% PFA for 10 min. After three washes in PBS, antigen retrieval was performed using a DAKO antigen retrieval buffer (DAKO Omnis, Agilent Technologies, Sata Clara, CA, USA), according to the manufacturer’s instructions. The slides were then allowed to cool at room temperature (RT) and washed in phosphate buffered saline (PBS). Permeabilization was performed with 0.3% Triton X-100 in PBS for 10 min, followed by blocking with 10% normal goat serum in 0.3% Triton X-100/PBS for 1 h at room temperature.

Sections were incubated overnight at 4 °C with primary antibodies targeting myelin proteolipid protein (PLP) (cat#ab28486, #ab9311, Abcam, Waltham, MA, USA, 1:200 dilution), glial fibrillary acid protein (GFAP) (cat#Z0334, DAKO Omnis, Agilent Technologies, Sata Clara, CA, USA, 1:300 dilution), ionized calcium binding adaptor molecule 1 (Iba1) (cat# 019-19741, FUJIFILM Wako Pure Chemical, Osaka, Japan, 1:200 dilution), CD68 (cat#ab53444, Abcam, Waltham, MA, USA, 1:200 dilution), sequestosome 1 (p62/SQSTM1) (cat#56416, Abcam, Waltham, MA, USA, 1:200 dilution) and ubiquitin (cat#7254, Abcam, Waltham, MA, 1:200 dilution). After washing, sections were incubated for 1 h at room temperature with appropriate Alexa Fluor 488 or Alexa Fluor 594 conjugated secondary antibodies (Thermal Fisher Scientific, Waltham, MA, USA, 1:500 dilution). The nuclei were counterstained with 4′,6-diamidino-2-phenylindole (DAPI). The stained sections were mounted with antifade medium and stored at 4 °C in the dark until imaging. Fluorescent images were captured using a Leica DM IL LED microscope (Leica, Wetzlar, Germany) for analysis.

### 2.6. X-Gal Histochemistry

The modified histochemical staining technique for the in situ localization of GALC activity was performed according to established protocols [15,16]. The cryosections were fixed in 4% paraformaldehyde for 15 min at RT and then equilibrated in citrate/phosphate buffer (C/P buffer, pH 4.2) for an additional 15 min at RT. The sections were then incubated with a solution containing taurodeoxycholic acid (TDCA, 5 mg/mL) and oleic acid (OA, 5 mg/mL) prepared in C/P buffer (pH 4.2). This was followed by staining with a X-Gal solution containing 2 mg/mL of X-Gal and 5 mM potassium ferricyanide/potassium ferrocyanide, which was supplemented with TDCA (5 mg/mL) and OA (5 mg/mL) in C/P buffer (pH 4.2) for 1.5 h at 37 °C. Finally, the sections were washed with phosphate buffered saline (PBS) and distilled water, followed by counterstaining with Nuclear Fast Red (Sigma-Aldrich, St Louis, MO, USA).

### 2.7. In Situ Hybridization

To confirm successful transfection of the AAV9-GALC vector into the brain, spinal cord, and sciatic nerve of Twi mice after gene therapy, GALC mRNA was detected using in situ hybridization with an Mm-GALC probe (cat#563541) designed via RNAscope™ technology (Advanced Cell Diagnostics, Newark, CA, USA).

The tissue cryosections mounted on slides were fixed in 4% paraformaldehyde pre-chilled in PBS at 4 °C for 15 min. After fixation, sections were sequentially dehydrated in graded ethanol solutions (50%, 70%, and 95%) for 5 min each at room temperature (RT) and air dried for 5 min. Using the RNAscope^®^ 2.5 HD Detection Kit–BROWN (cat#322310), endogenous peroxidase activity was quenched with RNAscope^®^ hydrogen peroxide. The slides were then treated with boiling 1X target recovery solution at 100 °C for 5 min, washed 3~5 times in distilled water at RT, rinsed in 100% ethanol, and air dried. Subsequently, the slides were treated with RNAscope Protease IV and incubated at 40 °C for 30 min. After washing with distilled water, hybridization was performed with the Mm-Galc probe at 40 °C for 2 h. The sections were washed with Wash Buffer for 2 min at RT and then sequentially hybridized with amplification reagents Amp1, Amp2, Amp3, Amp4, Amp5 and Amp6 for 30, 15, 30, 15, 30, and 15 min, respectively, with a 2-min Wash Buffer rinse at RT between each amplification step. The signal was detected using chromogenic DAB staining and the sections were counterstained with hematoxylin. Bright field microscopy was used to visualize the results.

### 2.8. Quantification of Viral Genomes by ddPCR

Quantification of AAV9 vector genome copies was performed using droplet digital PCR (ddPCR) on the Bio-Rad QX200 system (Bio-Rad Laboratories, Hercules, CA, USA) according to the manufacturer’s instructions. Duplex ddPCR reactions were prepared using primer–probe sets targeting the chicken β-actin (CBA) intron sequence within the AAV vector genome and the mouse *Rpp30* gene as a single-copy endogenous reference for normalization. Droplets were generated with the Bio-Rad Automated Droplet Generator, thermocycled on a T1000 Thermal Cycler, and subsequently read on the QX200 Droplet Reader. Vector genome (VG) copies per diploid genome (VG/DG) were calculated as the ratio of AAV CBA intron copies to *Rpp30* copies divided by two, providing precise quantification of AAV9 biodistribution in mouse brain tissues following CNS delivery.

### 2.9. Transmission Electron Microscopy

Sciatic nerves were prepared for transmission electron microscopy following standard protocols. Briefly, the samples were immersed in a fixative solution containing 2.5% glutaraldehyde and 1% osmium tetroxide, then dehydrated through a series of graded ethanol. Subsequently, the dehydrated samples were infiltrated with Spurr resin at three separate intervals of two hours each, culminating in polymerization at 70 °C for 8 h. The semi-thin sections (1 μm) were then cut from the embedded samples and stained with 0.5% toluidine blue to facilitate the identification of areas of interest under a light microscope. From these identified regions, ultrathin sections (70~90 nm) were prepared and subjected to staining with 2% methanolic uranyl acetate, followed by Reynolds lead citrate. The stained ultrathin sections were subsequently examined using a transmission electron microscope (JEM-1200EXII, JEOL Co., Tokyo, Japan) to analyze the ultrastructural characteristics of the sciatic nerve tissue.

### 2.10. Survival and Phenotype

Untreated Twi mice and WT mice were designated as control groups for the assessment of lifespan and weight. Both AAV9-treated (AAV9-Twi) and untreated Twi mice were monitored under standard conditions and allowed to live freely until they reached the moribund stage. All mice were subjected to daily observations, with body weights recorded weekly.

Motor coordination, strength, and locomotor function were assessed using a battery of behavioral tests, including the wire maneuver, rod balance, pole test, negative geotaxis, and wire hang test, as previously described [17,18,19]. For the wire maneuver, mice were suspended by the tail and lowered onto a horizontal wire. Scoring was as follows: 0, mouse swung hind legs to grasp the wire; 1, grasped the wire with struggling; 2, unable to grasp with hind limbs; 3, fell within 3 s; 4, fell immediately. The balance rod was used to test balance and motor and locomotor function. The mouse was placed on top and in the middle of a wooden rod (90 cm in length and 1.2 cm in diameter) that was 24 cm above the surface. Scores were assigned as follows: 0, traversed rod to end; 1, fell before reaching end; 2, froze for 60 s; 3, fell within 3 s; 4, fell immediately. The pole test was performed with minor modifications from Matsuura et al. [19] and measured strength and motor and locomotor function. The mice were placed head-up on a vertical pole and given 60 s to descend. Scores were assigned as follows: 0, turned and climbed down within 10 s; 1, took longer than 10 s; 2, turned but slid down; 3, turned and fell; 4, held on for 60 s; 5, held on or more than 30 s; 6, held on or more than 5 s; 7, fell immediately. The wire hang test provided a simple measure of strength. Latency to fall from an inverted cage lid, 50 cm above the surface, onto the soft bedding was recorded (maximum time 60 s).

### 2.11. Statistical Analysis

All results were expressed as the mean ± SD, and analyzed using the GraphPad Prism 6.0 software package. The survival rates between groups were plotted and analyzed using the Kaplan-Meier method, with a *p*-value of less than 0.05 considered statistically significant.

## 3. Results

### 3.1. Survival and Weight and Behavioral Assessment

**Figure 1:**
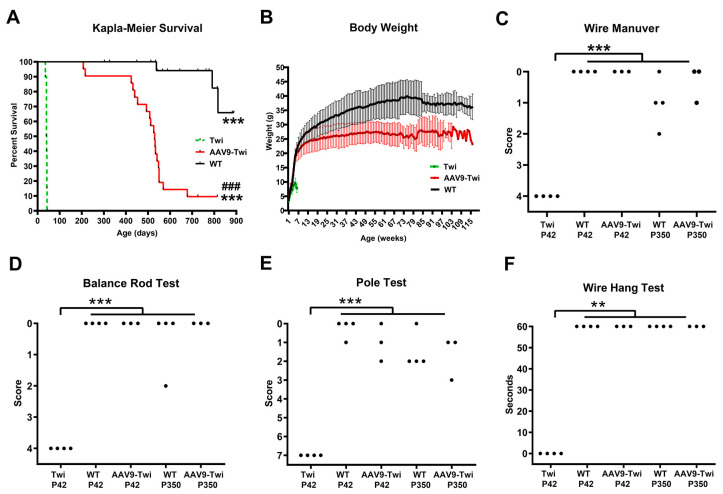
AAV9 Treatment Extends Survival and Improves Weight Gain and Behavioral Function in Twi Mice. (**A**) Kaplan-Meier survival curves demonstrate a significant extension of lifespan in AAV9-Twi mice (*n* = 22), reaching a maximum of 814 days, compared to untreated Twi mice (*n* = 16). WT mice (*n* = 16) are shown for reference. Vertical black ticks indicate censored animals, representing those removed at the study endpoint or for analysis. Statistical analysis was performed using Log-rank (Mantel-Cox) test. *** *p* < 0.001 compared to Twi, ### *p* < 0.001 compared to WT. (**B**) AAV9-Twi mice exhibit improved weight gain over time, in contrast to severely impaired growth in untreated Twi mice. Mean body weights for WT (*n* = 16), untreated Twi (*n* = 16), and AAV9-Twi mice are presented. (**C**–**F**) Behavioral assessments at P42 and P350 reveal that AAV9-Twi mice (*n* = 3) perform comparably to WT mice (*n* = 4) in wire maneuver, balance rod, pole, and wire hang tests. Untreated Twi mice display severe deficits at P42. Statistical analysis was performed using one-way ANOVA. ** *p* < 0.01; *** *p* < 0.001.

Untreated Twi mice exhibited a lifespan between 35 and 43 days, serving as a baseline for comparison with AAV9-treated twitcher (AAV9-Twi) mice. Administration of AAV9-delivered gene therapy resulted in a marked extension of survival, with treated Twi mice demonstrating a minimum lifespan of 207 days and a median survival of 530 days (Figure 1A). Notably, 90.5% of AAV9-treated twitcher mice survived beyond one year, and 66.7% attaining an advanced age exceeding 500 days. The two longest-lived AAV9-Twi mice survived for 726 and 814 days, respectively, before being euthanized to conclude the study.

The body weight of the experimental animals was recorded weekly and analyzed to assess the efficacy of the treatment (Figure 1B). The improvement benefit of AAV9-mediated gene therapy was also evident in terms of body weight. Untreated Twi mice exhibited a significant delay in weight gain beginning at P21, followed by progressive weight loss after P35, in contrast to WT and AAV9-Twi mice. Although AAV9-Twi mice displayed significantly lower body weight than WT mice after P42, their overall pattern of weight gain closely paralleled that of WT controls. In adulthood, AAV9-Twi mice reached a maximum body weight corresponding to approximately 75% of that observed in WT mice. Furthermore, AAV9-Twi mice preserved ambulatory function (Video S1) and feeding behavior throughout their lifespan, with only moderate weight loss observed prior to sudden death. Notably, two longest-lived AAV-Twi mice, surviving 726 and 814 days respectively, remained active and asymptomatic, displaying phenotypes comparable to age-matched WT mice. No age-related complications or late-onset adverse effects were observed in long-lived AAV9-Twi mice.

Functional assessments at P42 and P350 demonstrated preserved motor coordination, muscular strength, and locomotor activity in AAV9-Twi mice, as evaluated by wire maneuver (Figure 1C), rod balance (Figure 1D), pole test (Figure 1E), and wire hang assays (Figure 1F). Their performance was comparable to age-matched WT controls. In contrast, untreated Twi mice at P42 displayed profound impairments across these behavioral domains. Furthermore, AAV-Twi mice demonstrated normal spontaneous behaviors, including active locomotion (Video S1), drinking, and food consumption, indicative of sustained overall health and functional preservation following gene therapy. Normal behavior were supported by quantitative, validated behavioral assays and supplemental video documentation.

### 3.2. Supraphysiological Levels of GALC Activity

**Figure 2:**
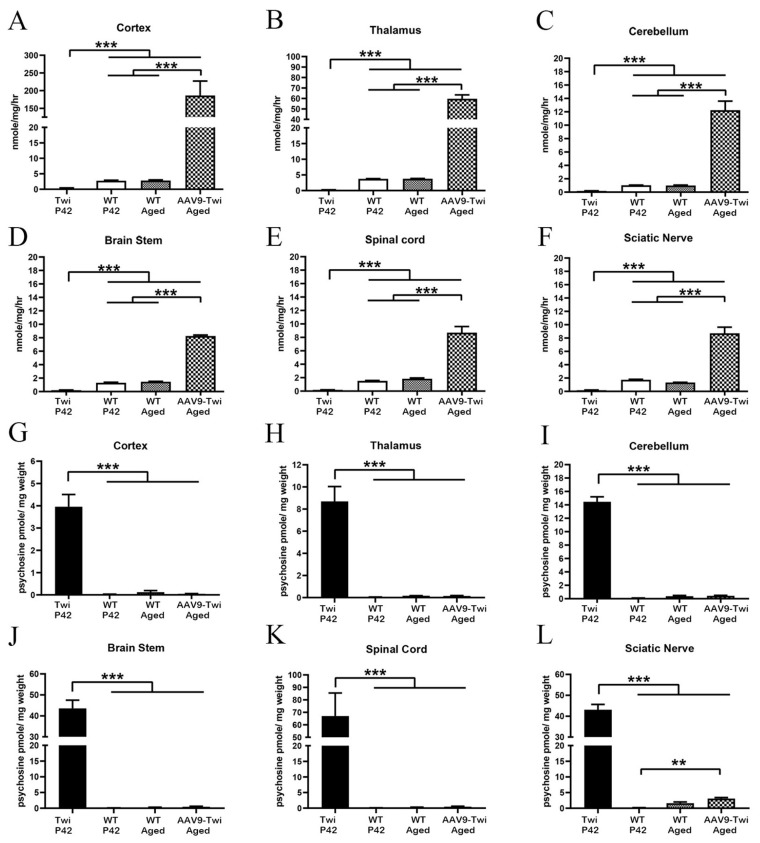
Supra-normal GALC Activity and Normalization of Psychosine Levels Following AAV9 Treatment in Twi Mice. (**A**–**F**) Aged (>P500) AAV9-Twi mice (*n* = 3) exhibit supra-normal GALC activity in the cortex, thalamus, cerebellum, brainstem, spinal cord, and sciatic nerve compared to P42 and aged (>P500) WT mice (*n* = 3 per group). In contrast, untreated Twi mice (*n* = 3, P42) display markedly reduced GALC enzymatic activity across these regions. (**G**–**L**) Psychosine concentrations in the cortex, thalamus, cerebellum, brainstem, spinal cord, and sciatic nerves are normalized in aged AAV9-Twi mice, reaching levels comparable to those observed in WT controls. Untreated Twi mice display substantial psychosine accumulation in all regions examined. The same tissue samples were used to measure GALC enzymatic activity in duplicate and psychosine levels in triplicate. Data are presented as mean ± SD. Statistical significance was determined by one-way ANOVA. ** *p* < 0.01; *** *p* < 0.001.

GALC activity was evaluated in multiple regions of the brain, including the cortex (Figure 2A), thalamus (Figure 2B), cerebellum (Figure 2C), brainstem (Figure 2D), spinal cord (Figure 2E), and sciatic nerve (Figure 2F) at P42 in near-moribund Twi mice and age-matched WT controls, as well as in aged (>P500) WT mice and aged (>P500) AAV9-Twi mice (Figure 2A–F). Tissue samples from Twi mice exhibited minimal detectable GALC activity compared to that of WT mice. GALC activity levels in aged WT mice were comparable to those observed at P42, indicating stable enzyme expression with age.

Remarkably, brain tissue from aged AAV9-Twi mice exhibited supranormal GALC activity relative to aged WT controls. In regions corresponding to AAV injection sites, GALC activity in the thalamus and cerebellum increased 20-fold and 12-fold, respectively, of the levels observed in aged wild-type mice, indicating efficient and robust transduction of AAV-GALC. Adjacent regions, including the cortex and brainstem, exhibited increases in GALC activity of up to 65-fold and 5.6-fold, respectively, relative to aged WT mice. Notably, distal regions such as the spinal cord and sciatic nerves also exhibited substantial increases, with GALC activity elevated approximately 4.5-fold and 6.5-fold, respectively, compared to aged WT mice. These findings of widespread, supranormal GALC activity in both CNS and PNS of AAV9-Twi mice indicate stable, robust, and sustained expression of AAV-delivered GALC throughout the life of the treated animals.

### 3.3. Normalization of Psychosine Concentration

In untreated Twi mice, lack of functional GALC leads to psychosine accumulation across tissues, notably in myelin-rich regions. To assess the relevance of GALC activity in catalyzing psychosine degradation, we measured psychosine levels in the same tissue samples used for the GALC activity assay. Mass spectrometry-based quantification revealed profound psychosine accumulation in the cortex (Figure 2G), thalamus (Figure 2H), cerebellum (Figure 2I), brainstem (Figure 2J), spinal cord (Figure 2K), and sciatic nerve (Figure 2L) of untreated Twi mice at P42, with the spinal cord exhibiting the highest levels. P42 and aged WT mice exhibited substantially lower psychosine levels across these regions (Figure 2G–L). Notably, aged AAV9-Twi mice (Figure 2G–L) showed psychosine concentrations in the cortex, thalamus, cerebellum, brainstem, and spinal cord comparable to those of P42 and aged WT mice. While psychosine levels in the sciatic nerve of AAV9-Twi mice were moderately elevated relative to P42 WT mice, they did not differ significantly from aged WT controls. These results demonstrate that AAV9-mediated GALC gene therapy effectively normalizes psychosine accumulation in both central and peripheral nervous system tissues, sustaining near-physiological levels throughout the lifespan of treated animals.

### 3.4. Intense and Broad GALC Expression

**Figure 3:**
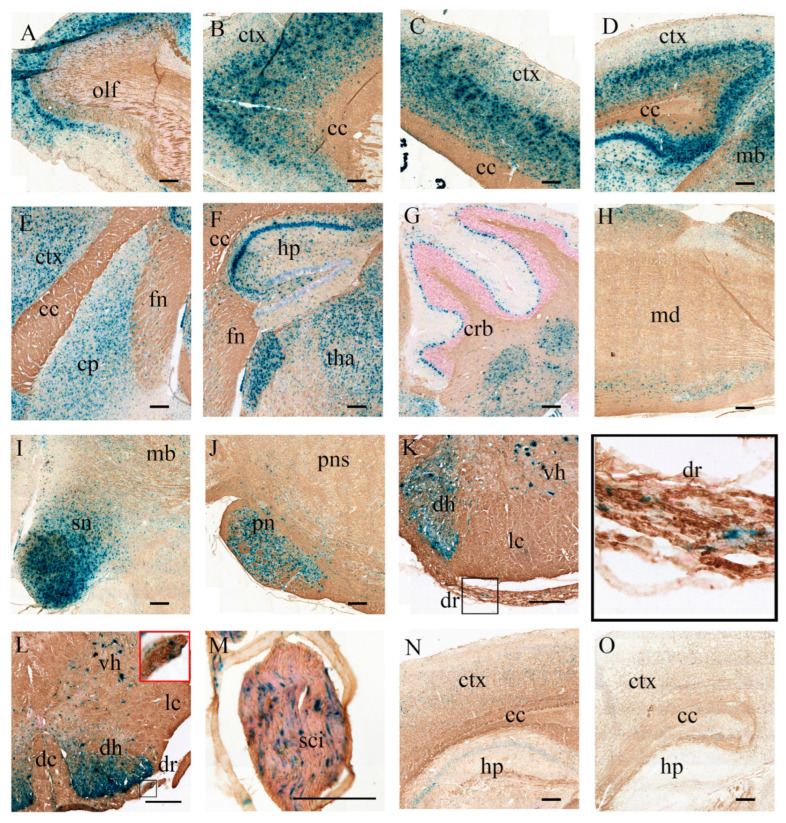
Widespread GALC Enzymatic Activity in the CNS and PNS of AAV9-Twi Mice. Modified X-Gal histochemical staining reveals robust GALC enzymatic activity throughout multiple regions of the central and peripheral nervous systems in aged (>P500) AAV9-Twi mice. Representative images show intense X-Gal staining in the (**A**) olfactory bulb; (**B**) prefrontal cortex; (**C**) cingulate cortex; (**D**) retrosplenial cortex; (**E**) caudate putamen; (**F**) hippocampus and thalamus; (**G**) cerebellar white matter and Purkinje cells; (**H**) medulla; (**I**) substantia nigra; (**J**) pons; dorsal and ventral horns of the (**K**) cervical and (**L**) lumbar spinal cord, including a higher magnification of intense GALC expression in the dorsal root (inset), respectively; and (**M**) longitudinal section of the sciatic nerve. Note the sparse X-Gal staining observed in the corpus callosum, fornix, and dorsal and lateral columns of the spinal cord. Cortical sections from (**N**) P42 WT and (**O**) P42 untreated Twi mice are shown as controls. Scale bars: 200 μm. Abbreviations: cc, corpus callosum; cp, caudate putamen; crb, cerebellum; ctx, cortex; dc, dorsal column; dh, dorsal horn; dr, dorsal root; fn, fornix; hp, hippocampus; lc, lateral column; mb, midbrain; md, medulla; olf, olfactory bulb; tha, thalamus; sn, substantia nigra; pns: pons; pn, pontine nucleus; vh, ventral horn; sci, sciatic nerve.

To visualize GALC enzymatic activity following AAV-mediated gene therapy, a modified X-Gal histochemical staining protocol was employed, as previously described, which selectively inhibits endogenous β-galactosidase activity to enable specific in situ detection of GALC activity in the CNS and PNS (Figure 3) [15].

In aged AAV9- Twi mice (Figure 3A–M), intense X-Gal staining was observed at the primary injection sites, including the thalamus and cerebellar white matter, corresponding to localized GALC enzymatic activity. Notably, robust and widespread staining extended from the olfactory bulb (Figure 3A), rostral infralimbic cortex (Figure 3B), cingulate cortex (Figure 3C), to caudal retrosplenial granular cortex and midbrain (Figure 3D). Intense staining was also detected in caudate putamen (Figure 3E), thalamus (Figure 3F) and hippocampus (Figure 3F). Within the hippocampus (Figure 3F), strong X-Gal signals were detected in neurons of the CA1, CA2, CA3 regions, and the dentate gyrus. Prominent staining was also observed in cerebellar white matter and Purkinje cells (Figure 3G).

In the brainstem and midbrain, variable staining intensities were detected in medulla (Figure 3H), with particularly strong signal in the substantia nigra (Figure 3I) and basilar pontine nuclei (Figure 3J). In sagittal spinal cord sections, abundant staining was evident along both the dorsal and ventral horns, and dorsal root of cervical (Figure 3K) and lumbar spinal cord (Figure 3L), and sciatic nerve (Figure 3M). Of note, X-Gal staining was also observed in the corpus callosum (Figure 3B–E), fornix (Figure 3E,F), and dorsal and lateral columns of the spinal cord (Figure 3K,L).

In WT mice at P42, X-Gal staining was evident in neurons of the cortex (Figure 3N), hippocampus (Figure 3N), and Purkinje cell layer. Moderate staining was also evident observed in the corpus callosum, mossy fibers of the caudate putamen, and cerebellar white matter. In contrast, no detectable X-Gal staining was present in the corresponding brain regions of untreated Twi mice (Figure 3O), consistent with the loss of GALC enzymatic function.

Collectively, our findings indicate widespread and sustained GALC expression following AAV9-mediated gene delivery, supporting effective transduction and enzymatic activity across multiple neuroanatomical regions of the CNS and PNS.

### 3.5. Sustained and Widespread Biodistribution

We used RNAscope technology, a highly sensitive and specific in situ hybridization method, to detect the delivery of therapeutic DNA molecules mediated by AAV9-based CNS-targeted gene therapy in the CNS and PNS of Twitcher mice. RNAscope utilizes target-specific probes to hybridize with RNA transcripts of interest, enabling single-molecule detection through a branched amplification system that generates intense and localized chromogenic signals.

**Figure 4:**
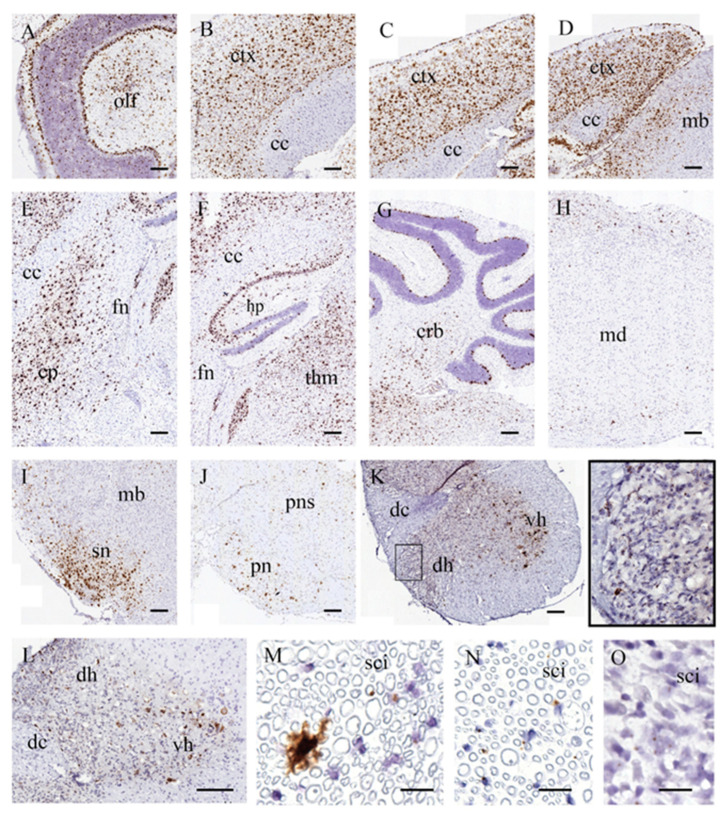
Sustained and Widespread Biodistribution in the CNS and PNS of AAV9-Twi Mice. Spatial biodistribution of AAV9-delivered GALC was assessed by in situ hybridization. Robust GALC mRNA expression is detected across multiple regions of the central and peripheral nervous systems in aged AAV9-Twi mice. Representative images show intense hybridization signals in the (**A**) olfactory bulb; (**B**) prefrontal cortex; (**C**) cingulate cortex; (**D**) retrosplenial cortex; (**E**) caudate putamen; (**F**) hippocampus and thalamus; (**G**) cerebellar white matter and Purkinje cells; (**H**) medulla; (**I**) midbrain; (**J**) pons; dorsal and ventral horns of the (**K**) cervical, including a higher magnification of the intense hybridization signals in the dorsal horn (inset), and (**L**) lumbar spinal cord; and (**M**) cross section of the sciatic nerve. Sciatic nerve sections from (**N**) P42 WT and (**O**) P42 untreated Twi mice are included for comparison. Scale bars: 200 μm (**A**–**L**), 20 μm (**M**–**O**). Abbreviations: cc, corpus callosum; cp, caudate putamen; crb, cerebellum; ctx, cortex; dc, dorsal column; dh, dorsal horn; dr, dorsal root; fn, fornix; hp, hippocampus; lc, lateral column; mb, midbrain; md, medulla; olf, olfactory bulb; tha, thalamus; sn, substantia nigra; pns: pons; pn, pontine nucleus; vh, ventral horn; sci, sciatic nerve.

Using an RNAscope probe designed for mGALC mRNA, the hybridized signals were amplified and visualized as chromatic puncta, representing individual RNA molecules (Figure 4).

Robust and highly localized signals were detected in the brains of aged AAV9-Twi mice, demonstrating effective transgene delivery and expression (Figure 4A–M). Notably, the olfactory bubs (Figure 4A), rostral infralimbic cortex (Figure 4B), cingulate cortex (Figure 4C), caudal retrosplenial granular cortex (Figure 4D), midbrain (Figure 4D), caudate putamen (Figure 4E), and thalamus (Figure 4F) exhibited a broad and dense distribution of intense chromogenic signals. In the hippocampus (Figure 4F), strong signals were detected in the pyramidal cell layers of CA1, CA2, and CA3, as well as in the hilus and granular cell molecular layers of the dentate gyrus. Puncta signals were also observed in the corpus callosum (Figure 4B–F) and fornix (Figure 4E,F).

In the cerebellum (Figure 4G), prominent signals were localized to the Purkinje cell layer and white matter. Furthermore, intense signals were widely distributed in the medulla (Figure 4H), substantia nigra (Figure 4I), pons (Figure 4J), dorsal horn and ventral horn of cervical (Figure 4K) and lumbar (Figure 4L) spinal cord. Intriguingly, intense puncta signals were detected in the sciatic nerve (Figure 4M), underscoring the extensive reach of AAV9-mediated transgene delivery.

In comparison, clear and intense GALC transcript puncta were observed in sciatic nerve of WT mice (Figure 4N), while sciatic nerve from untreated Twitcher mice exhibited weakly stained GALC puncta and loss of myeline (Figure 4O).

These findings demonstrate that CNS-targeted AAV9 gene therapy facilitated widespread and robust delivery of therapeutic DNA, extending rostrally to the olfactory bulbs and caudally to the sciatic nerve. The use of the RNAscope provided critical insights into the spatial distribution and expression patterns of the transgene, underscoring its utility in evaluating the efficacy of gene therapy.

**Figure 5:**
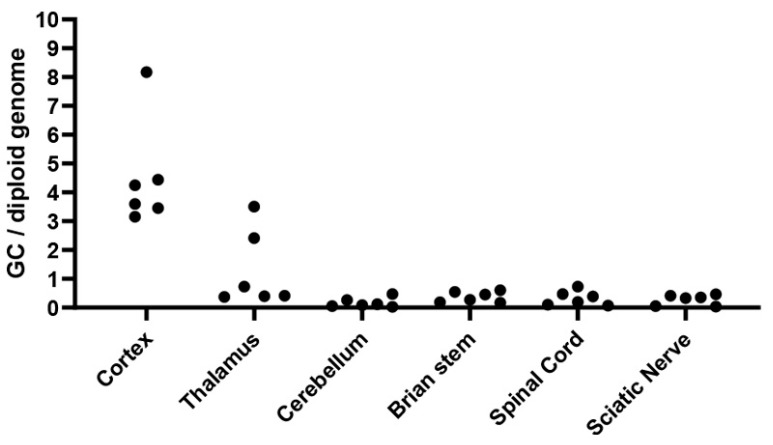
Quantification of Vector Genome Biodistribution in the Nervous System of Aged AAV9-Twi Mice. Vector genome copies were quantified by droplet digital PCR (ddPCR) in multiple regions of the nervous system from aged AAV9-treated Twitcher (AAV9-Twi) mice (*p* > 500; *n* = 3; measured in duplicate). Quantitative data are normalized to the endogenous *Rpp30* reference gene and expressed as vector genome (VG) copies per diploid genome.

Consistent with the in situ hybridization results, quantitative analysis of viral vector biodistribution (Figure 5) revealed the highest AAV9 transduction in the cortex (3.15–8.17 GC/DG), followed by the thalamus (0.37–3.50 GC/DG), brainstem (0.17–0.60 GC/DG), spinal cord (0.07–0.70 GC/DG), cerebellum (0.03–0.47 GC/DG), and sciatic nerve (0.03–0.46 GC/DG). These findings corroborate the broad yet regionally graded distribution of AAV9-mediated GALC expression observed by in situ hybridization, reflecting efficient transduction in both central and peripheral nervous system compartments.

### 3.6. Reduction of Neuroinflammation

Neuroinflammation and demyelination constitute hallmark pathological features of GLD. In twitcher mice, progressive activation of microglia, astrocytosis, and macrophage infiltration occurs in a spatiotemporal pattern, initially affecting the cerebellar white matter and brainstem after P20, subsequently involving the cerebral white matter after P25, and extending to the cerebral gray matter by P30 [20,21]. To evaluate the efficacy of AAV9-GALC gene therapy in mitigating neuroinflammation, immunohistochemical analyses were performed targeting astrocytes and microglia using GFAP and Iba-1 markers, respectively.

**Figure 6:**
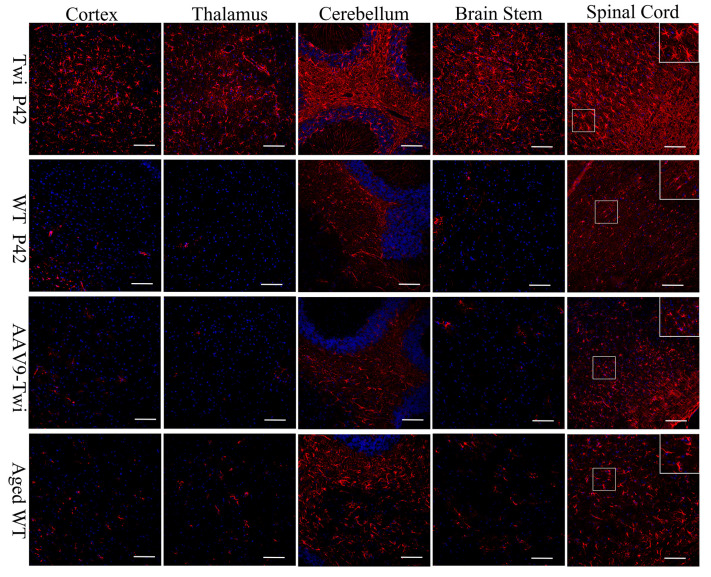
Reduction of astrocyte activation in the brain and spinal cord of aged AAV9-Twi mice. Immunohistochemical staining for glial fibrillary acidic protein (GFAP) with DAPI counterstaining reveals pronounced astrocyte activation in the cortex, thalamus, cerebellum, brain stem, and spinal cord of P42 Twi mice compared to P42 and aged (>P500) WT controls. Aged WT mice exhibit a moderate increase in astrocyte presence within the cerebellum and spinal cord relative to P42 WT mice. Aged (>P500) AAV9- Twi mice display a similar moderate level of astrocyte presence in these regions, comparable to aged WT controls, reflecting age-related gliosis and preserved glial homeostasis in the central nervous system following AAV9-mediated gene therapy. Insets in the spinal cord panels illustrate a higher magnification of hypertrophic reactive astrocytes in Twi, and resting astrocytes in WT, aged WT, and aged AAV9-Twi mice. Scale bars, 200 μm.

At P42, Twi mice exhibited extensive reactive astrogliosis in the corpus callosum, cerebellar white matter, brainstem, and spinal cord, characterized by hypertrophic somas and thick stellate processes (Figure 6). In contrast, age-matched WT mice showed sparse, resting astrocytes with small somas and thin processes in the corpus callosum and cerebellar white matter (Figure 6). While aged WT mice displayed increased astrocytic density in these regions, most astrocytes remained in a resting state (Figure 6). Notably, aged AAV9-Twi mice demonstrated reduced astrocytic burden in the brain and spinal cord, predominantly composed of resting astrocytes, compared to aged WT mice (Figure 6).

**Figure 7:**
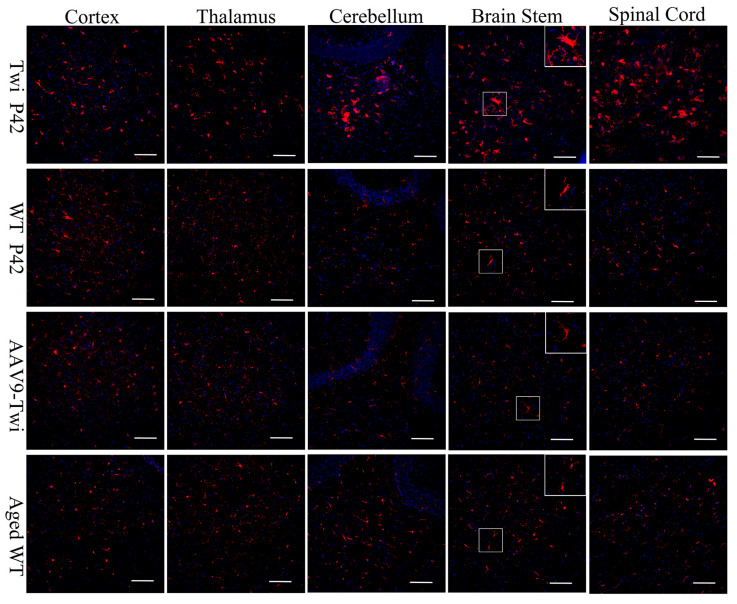
Inhibition of Globoid Cell Activation in the Brain and Spinal Cord of Aged AAV9-Twi Mice. Untreated Twi mice at P42 exhibit pronounced microglial activation and globoid cell formation in the cortex, thalamus, cerebellum, brainstem, and spinal cord, as demonstrated by immunohistochemical staining for ionized calcium-binding adapter molecule 1 (Iba1) with DAPI counterstaining, compared to P42 and aged (>P500) WT controls. Aged (>P500) AAV9-Twi mice exhibit markedly reduced Iba1+ staining and an absence of globoid cell clusters, comparable to levels observed in P42 and aged WT mice, indicating effective suppression of microglial activation and prevention of neuroinflammatory pathology following AAV9-mediated gene therapy. Insets in the brainstem panels illustrate a higher magnification of reactive microglia and globoid cells in Twi, and resting microglia in WT, aged WT, and aged AAV9-Twi mice. Scale bars, 200 μm.

Microglial activation, a key contributor to multinucleated globoid cell formation and demyelination in GLD, was also evaluated [22,23]. At P42, the majority of microglia in the brains of WT mice remained in resting state, characterized by small somas and highly ramified processes (Figure 7). In contrast, untreated Twi mice exhibited a significant increase in reactive microglia, particularly in the brain stem and spinal cord (Figure 7). These microglia displayed hypertrophic somas with thickened bushy processes, along with amoeboid-like and multinucleated globoid cells, indicative of a highly activated phagocytic state (Figure 7). In both aged WT and AAV9-Twi mice, microglia largely retained resting morphologies, with only a moderate increase in hypertrophic microglia compared to younger WT mice, consistent with an ageing-associated low-grade pro-inflammatory state (Figure 7). The substantial reduction in neuroinflammation observed in AAV9-Twi mice at advanced age indicates the durable efficacy of gene therapy.

**Figure 8:**
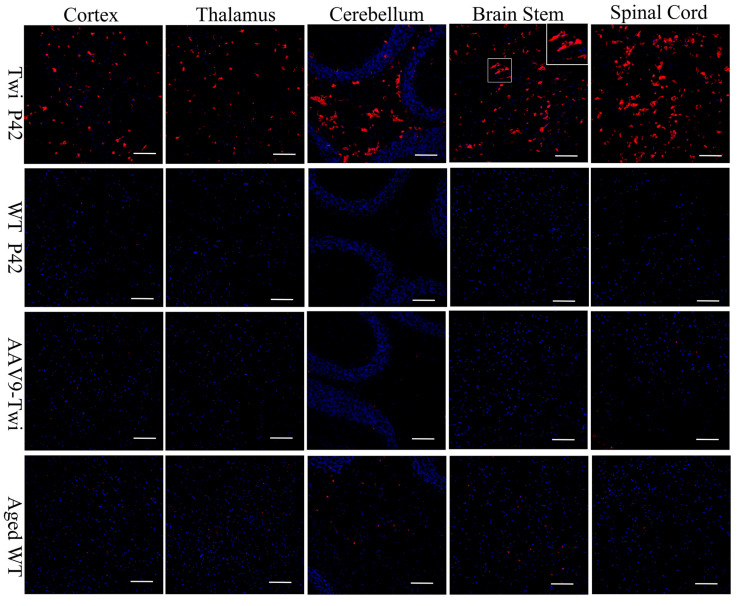
Ablation of Macrophage/Microglia Infiltration in the Brain and Spinal Cord of Aged Twi Mice Following AAV9 Treatment. Untreated Twi mice at P42 exhibit pronounced macrophage/microglia infiltration in the cortex, thalamus, cerebellum, brainstem, and spinal cord, as demonstrated by immunohistochemical staining for CD68 with DAPI counterstaining, compared to P42 and aged (>P500) WT controls. In contrast, aged (>P500) AAV9-Twi mice show an absence of macrophage/microglia infiltration in these regions, comparable to that observed in P42 and aged WT mice, indicating effective suppression of neuroinflammation and innate immune activation following AAV9-mediated gene therapy. Inset in the brainstem panel illustrates a higher magnification of morphology of CD68-positive macrophagy/microglia in Twi. Scale bars, 200 μm.

Previous investigations have shown that CD68+ macrophage/microglia infiltration correlates with the progression of GLD disease and the severity of demyelination, occurring concomitantly with astrogliosis [24,25]. In the present study, immunohistochemical analyses revealed pronounced infiltration of CD68+ macrophages/microglia within the brain and spinal cord parenchyma of untreated Twi mice (Figure 8). In contrast, CD68 immunoreactivity was absent in both wild-type and aged AAV9-Twi mice, signifying effective suppression of neuroinflammatory responses through therapeutic GALC restoration (Figure 8). These findings underscore the pivotal role of CD68+ cells in GLD pathogenesis and highlight the capacity of AAV9-mediated gene therapy to modulate pathogenic immune activation.

### 3.7. Preservation of Proteostasis

**Figure 9:**
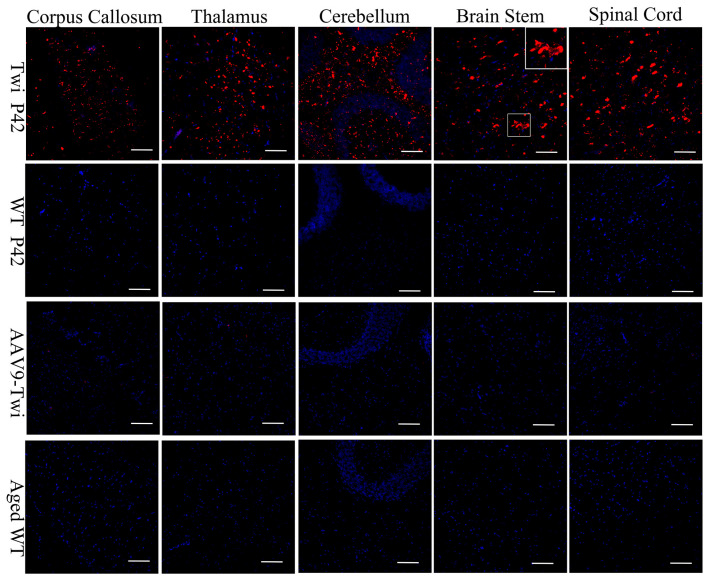
Preservation of Autophagy Function in Brain and Spinal Cord of Aged AAV9-Twi Mice. Immunohistochemical staining for p62 with DAPI counterstaining reveals extensive accumulation of p62-positive aggregates in the cortex, thalamus, cerebellum, brainstem, and spinal cord of untreated Twi mice at P42, compared to P42 and aged (>P500) WT controls. In contrast, aged (>P500) AAV9-treated Twi mice show an absence of p62 aggregates in these regions, comparable to that observed in P42 and aged WT mice, indicating preserved autophagy function and proteostasis in the nervous system following AAV9-mediated gene therapy. Inset in the brainstem panel illustrates a higher magnification of p62-aggregates in Twi. Scale bars, 200 μm.

**Figure 10:**
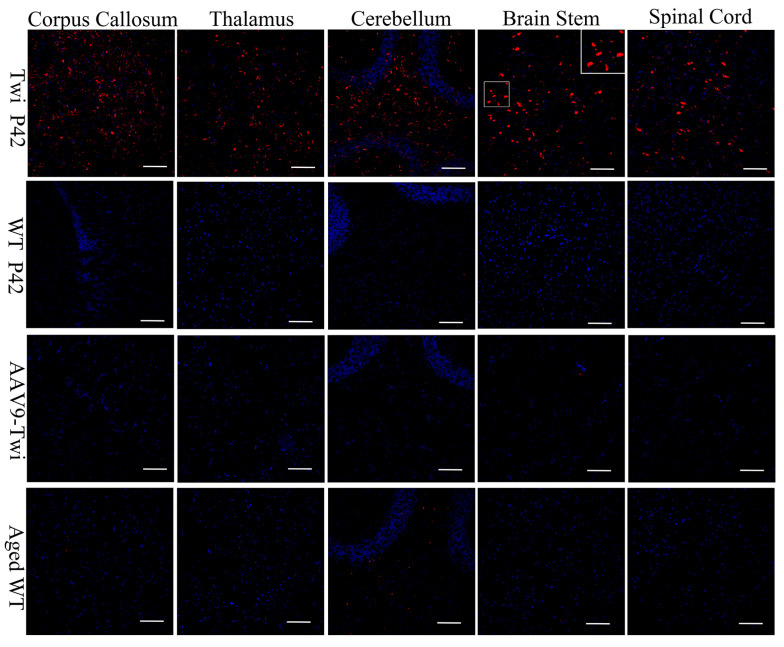
Preservation of the Ubiquitin-Proteasome System in the Brain and Spinal Cord of Aged AAV9-Twi Mice Following AAV9 Treatment. Immunohistochemical staining for ubiquitin with DAPI counterstaining reveals extensive accumulation of ubiquitin-positive aggregates in the cortex, thalamus, cerebellum, brainstem, and spinal cord of untreated Twi mice at P42, compared to P42 and aged (>P500) WT controls. In contrast, aged (>P500) AAV9-Twi mice exhibit an absence of ubiquitin-positive aggregates in these regions, comparable to that observed in P42 and aged WT mice, indicating restoration of protein homeostasis and preservation of ubiquitin–proteasome system function in the nervous system following AAV9-mediated gene therapy. Inset in the brainstem panel illustrates a higher magnification of ubiquitin-positive aggregates in Twi. Scale bars, 200 μm.

The efficacy of AAV9-mediated GALC gene therapy in restoring autophagy and UPS function in the CNS of twitcher mice was evaluated. Psychosine accumulation has been shown to impair autophagy and UPS in oligodendrocytes, leading to progressive aggregation of misfolded proteins in white matter regions [4,5,24]. In the current study, the therapeutic efficacy of AAV9-GALC was assessed by immunohistochemical detection of p62 (Figure 9) and ubiquitin-positive aggregates (Figure 10). At P42, untreated Twi mice exhibited a wide distribution of p62- (Figure 9) and ubiquitin-positive (Figure 10) aggregates throughout the brain, with the greatest burden localized to the spinal cord, brainstem, cerebellum, and corpus callosum. In contrast, P42 wild-type mice, aged WT controls, and aged AAV9-Twi mice exhibited no detectable aggregates in the brain or spinal cord (Figure 9 and Figure 10). These results indicate that AAV9-GALC gene therapy effectively restores proteostasis and prevents aggregate accumulation, highlighting its therapeutic potential in mitigating autophagy and UPS dysfunction in GLD.

### 3.8. Preservation of Myelination and Axon Integerity

**Figure 11:**
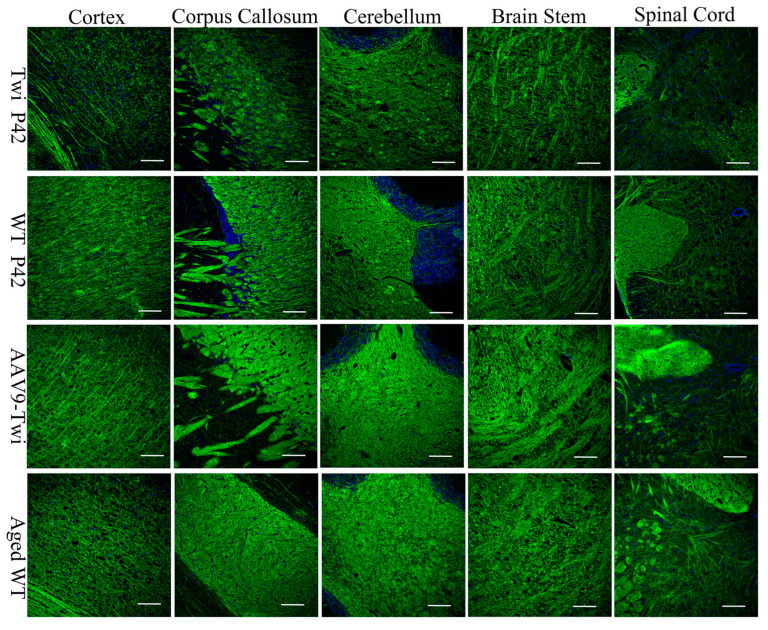
Preservation of Myelination in the Brain and Spinal Cord of Aged AAV9-Twi Mice. Myelination was evaluated by proteolipid protein (PLP) immunostaining with DAPI counterstaining in brain and spinal cord sections from P42 WT mice, P42 Twi mice, aged WT mice (>P500), and aged AAV9-Twi mice (>P500). AAV9 treatment results in sustained preservation of myelination throughout the brain and spinal cord of aged AAV9-Twi mice, comparable to that observed in aged WT controls. In contrast, untreated Twi mice at P42 exhibit severe demyelination in subcortical white matter, corpus callosum, cerebellar white matter, brainstem, and spinal cord. Scale bars, 200 µm.

Demyelination in twitcher mice typically begins between P15–P20, initially affecting the cerebellar white matter and brainstem, and progressively extending to the cerebral white matter, spinal cord, sciatic nerve, and eventually gray matter regions [20,26,27]. To assess the therapeutic efficacy of AAV9-mediated gene therapy in preserving CNS myelination, immunofluorescent histochemical analysis was performed. In untreated Twi mice at P42, extensive demyelination was evident across multiple CNS regions, including the subcortical white matter, corpus callosum, cerebellar white matter, brainstem, and spinal cord (Figure 11). In contrast, aged AAV9-Twi mice exhibited robust and widespread preservation of myelination throughout the brain and spinal cord, closely resembling the myelination patterns observed in age-matched WT controls (Figure 11).

**Figure 12:**
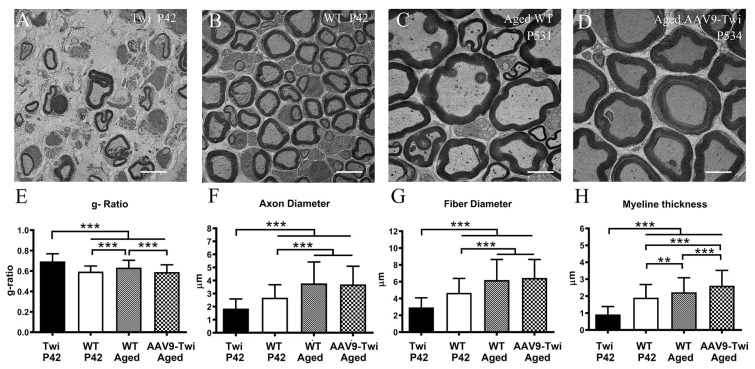
Correction of axonopathy in the sciatic nerves of aged AAV9-Twi mice. AAV9-mediated gene therapy preserves normal axonal morphology and myelination in the sciatic nerve (SN) of aged AAV9-treated Twi mice. Transmission electron microscopy (TEM) images reveal profound axonal degeneration and demyelination in the SN of (**A**) P42 Twi mice compared to (**B**) P42 WT and (**C**) aged WT mice. (**D**) Aged AAV9-treated Twi mice exhibit well-preserved myelination and intact axons comparable to those observed in aged WT controls. Quantitative analysis of myelinated axon structural integrity includes (**E**) g-ratio, (**F**) axon diameter, (**G**) fiber diameter, and (**H**) myelin thickness, measured from TEM images of SN samples from P42 Twi, P42 WT, aged WT (>P500), and aged (>P500) AAV9-treated Twi mice. AAV9 treatment results in sustained preservation of axonal integrity in the SN of aged Twi mice. Data represent mean ± SD derived from 150 axons per group, with *n* = 3 animals per group. Statistical significance was determined by one-way ANOVA. ** *p*  <  0.01; *** *p*  <  0.001. Scale bars, 5 μm.

To evaluate the impact of AAV9-GALC on PNS myelination, sciatic nerve ultrastructure was examined by transmission electron microscopy. Untreated Twi mice showed marked demyelination and axonal degeneration (Figure 12A), whereas aged AAV9-Twi mice exhibited well-preserved axonal morphology and compact myelin, resembling the WT phenotype (Figure 12B–D). Quantitative analysis of myelination based on g-ratio measurements revealed that P42 Twi mice had significantly reduced axon fiber diameter, axonal diameter, and myelin thickness, along with elevated g-ratio values, compared to WT, aged WT, and aged AAV9-Twi mice (Figure 12E–H). While aged WT mice showed increased g-ratios and axon dimensions relative to P42 WT mice, aged AAV9-Twi mice displayed normalized axon and fiber diameters, increased myelin thickness, and a g-ratio restored to levels observed in P42 WT mice.

Collectively, these findings demonstrate that AAV9-mediated GALC gene therapy effectively preserves CNS and PNS myelination and maintains axonal integrity in Twi mice.

## 4. Discussion

The multifaceted pathology of GLD, characterized by the involvement of both the CNS and the PNS, the accumulation of cytotoxic psychosine, profound neuroinflammation, impaired autophagy, and rapid progression of the disease, poses significant challenges for effective therapeutic intervention. Although gene therapy has demonstrated efficacy in alleviating symptoms and extending lifespan, prior monotherapy approaches utilizing viral vectors alone have been insufficient to comprehensively address all pathological aspects concurrently. The incorporation of HSCT, with or without SRT, has provided additional enzymatic and metabolic correction, further prolonging survival; nevertheless, achieving complete disease rescue remains an ongoing challenge. Importantly, the present study surpasses previous outcomes by demonstrating, for the first time, comprehensive pathological correction via minimal CNS-targeted monotherapy, providing compelling evidence of its potential as a standalone therapeutic strategy.

Early studies employing intracerebral administration of AAV1 (3 × 10^10^ vg) and AAV5 (2.4 × 10^9^ vg) in neonatal Twitcher mice modestly prolonged survival by 10 to 15 days, outperforming AAV2 and adenoviral vectors [17,28]. Previous work with AAV5 (2.4 × 10^9^ vg) delivery targeting the cortex, hippocampus and cerebellum extended the maximum survival to 66 days [29,30]. Subsequent refinements involving intrathecal (i.t.) and six-separate intracranial (i.c.) injections of AAV5 (3.6 × 10^10^ vg) further improved maximal survival to 78 days [31].

Advances in vector engineering introduced AAVrh10 and AAV9, characterized by a wider tropism of the CNS. Neonatal administration combining intracerebroventricular (i.c.v., 3 × 10^9^ vg), intracerebellar (1.5 × 10^9^ vg), and intravenous (i.v., 7.6 × 10^9^ vg) routes of AAVrh10 extended maximum survival to 240 days [32]. Further optimization with a tenfold increase in intravenous (i.v., 4 × 10^14^ vg/kg) dosing of AAVrh10 enhanced maximal survival 430 days [33]. Parallel studies employing AAV9 via 5-seperat i.c. (9 × 10^9^ vg), intrathecal (i.t., 8.25 × 10^10^ vg), and intravenous (i.v., 3.3 × 10^11^ vg) administration reported a maximum of 484 days [34].

To address limitations of single-modality gene therapy, combination strategies integrating AAV9-mediated CNS delivery (i.c. and i.t., 2.7 × 10^10^ vg) with HSCT and SRT achieved median survival of 404 days and maximum survival of 569 days [35]. Meanwhile, the combination of AAV9-mediated CNS administration (i.c. and i.t., 2.7 × 10^10^ vg) and HSCT alone extended median and maximal survival to 269 and 673 days, respectively [35]. Similarly, AAVrh10 i.v. (4 × 10^13^ vg/kg) administration in combination with HSCT further prolonged the median lifespan to 351 days, with a maximum survival of 750 days [33].

Previous studies (Table 1) employing multiple i.c. injections or combined CNS delivery routes (i.c., i.c.v., and i.t. administration) achieved only limited extension of lifespan in twitcher mice, primarily due to incomplete correction of both CNS and PNS pathology [17,28,29,30,31]. The addition of systemic i.v. AAV delivery (Table 1) improved outcomes by restoring GALC activity within the PNS and further prolonging survival [32,33,34]. Yet, the limited ability of i.v. AAV to cross blood-brain barrier constrained overall efficacy. When systemic i.v. high dose AAV delivery combined with HSCT, which mitigates neuroinflammation, this dual-therapy regimen (Table 1) achieved the longest survival previously reported [33]. Notably, our protocol with minimal and region-specific CNS delivery of high-titer AAV9 surpasses all prior benchmarks, achieving a median survival of 530 days and maximum survival of 814 days, approaching the WT lifespan. Critically, this streamlined approach circumvents the risks associated with HSCT, such as graft-versus-host disease, and avoids the complexities inherent to multi-route and multi-dose regimens, or high-dose systemic administration. The sustained therapeutic efficacy observed in long-lived animals further suggests that one-time treatment may confer a lifelong corrective effect. Those results underscore the transformative potential of our protocol as a standalone treatment for GLD, while remaining complementary to more complex multimodal strategies.

AAV9-mediated gene therapy has demonstrated substantial therapeutic efficacy in addressing pathological gene deficiencies, particularly in disorders affecting both the CNS and PNS. However, challenges persist in achieving spatially uniform transgene distribution, sustained expression, and adequate enzymatic activity in both the CNS and the PNS. In this study, intraparenchymal delivery of high-titer AAV9 targeting the thalamus and deep cerebellum resulted in widespread expression and activity of GALC throughout the brain, cerebellum, spinal cord, and sciatic nerves of aged AAV9-Twi mice. Histochemical staining confirmed robust transduction of neurons and glia across proximal CNS regions, as well as distal PNS tissues, facilitated by efficient anterograde and retrograde axonal transport mechanisms [37]. Notably, GALC activity reached supranormal levels in all neural compartments, with maximum activity observed in the cortex and thalamus, followed by elevated levels in the cerebellum, brainstem, spinal cord, and sciatic nerves. This extensive biodistribution underscores the ability of AAV9 to leverage axonal transport pathways for global transgene delivery without requiring multiple injection sites or invasive interventions. The advantages of AAV9 include its ability for efficient neuron/glia transduction, sustained enzymatic activity throughout the nervous system, and long-term therapeutic efficacy. Collectively, both thalamus and deep cerebellar nuclei are highly interconnected hubs with extensive projections to cortex, brainstem, and spinal cord [38,39], enabling robust anterograde and retrograde axonal transport of AAV9. Neonatal parenchyma also supports enhanced viral diffusion due to reduced myelination and greater extracellular permeability [40]. These mechanisms collectively explain how focal injections can yield widespread GALC expression without multi-route delivery. By obviating the need for systemic administration, multi-route, or multi-dose, current streamlined therapy offers a targeted yet comprehensive approach to addressing CNS/PNS pathologies while minimizing complexity of therapeutic approach and improving safety.

Psychosine accumulation due to GALC deficiency is central to the pathogenesis of GLD. The efficacy of AAV-mediated gene therapy in normalizing psychosine levels in Twitcher mice has been a critical focus in evaluating its therapeutic potential. Previous studies have reported incomplete normalization of psychosine within the nervous system following AAV-based interventions, whether administered alone or in combination with other therapies [31,34,35,41]. These limitations have been attributed to suboptimal AAV transduction efficiency, heterogeneous distribution of GALC activity in regions susceptible to residual psychosine accumulation, and insufficient GALC activity gradients to effectively counteract localized psychosine synthesis [34,35,41]. In the present study, for the first time in gene therapy of GLD, psychosine levels in both the CNS and the PNS of aged AAV9-treated Twitcher mice were comparable to those observed in aged wild-type mice. Importantly, these normalized levels remained stable throughout the lifespan of treated animals, indicating sustained transgene expression and adequate therapeutic GALC activity across all relevant CNS and PNS regions, thereby conferring durable therapeutic benefit. Furthermore, robust AAV9-GALC transduction was detected in CNS gray matter and spinal cord, consistent with AAV9 tropism, with additional transduction in white matter and sciatic nerves. These observations suggest that both local GALC production by transduced neurons and myelinating cells, as well as cross-correction from highly transduced neurons, contributed to supraphysiological GALC activity. Aligning with observations in previous long-term studies [33,34,41], the supraphysiological GALC levels were not associated histopathological or behavioral deficits throughout the extended longevity of AAV9-Twi mice. The sustained supranormal GALC activity ensures robust substrate clearance of psychosine and galactosylceramide in demyelination-prone regions over the life span, critical for preventing demyelination and neurodegeneration in Krabbe disease models. Collectively, these mechanisms underpin the global normalization of psychosine levels observed in AAV9-Twi mice.

In Twitcher mice, axonopathy precedes overt demyelination and neuronal loss, identifying axonal degeneration as an early and primary event in GLD pathogenesis [42]. Psychosine accumulation independently disrupts axonal transport and initiates dying-back neuropathy, effects that cannot be mitigated by glial GALC expression alone [36,42]. Additionally, Psychosine also directly impairs axonal transport and cytoskeletal organization, resulting in synaptic dysfunction and neurodegeneration [43,44]. These neurotoxic effects may persist despite remyelination if neuronal GALC activity is insufficient. Moreover, neuronal GALC deficiency leads to brainstem maldevelopment, axonal atrophy, neuroinflammation, and demyelination, underscoring its essential role in maintaining neuronal integrity [45]. In the present study, sustained psychosine clearance across CNS and PNS tissues of aged AAV9-treated Twi mice implicates effective correction in both neuronal and oligodendrocytic compartments. The preservation of compact myelin, normal axonal morphology in sciatic nerves, and restored motor behavior collectively confirm dual-compartment rescue. These results support that long-term therapeutic success in GLD requires robust GALC expression in both neurons and myelinating glia to prevent psychosine-mediated neurotoxicity and secondary demyelination.

Notably, the spatially heterogeneous distribution of transgenes in the CNS of Twitcher mice induces focal episomal vector depletion, allowing localized psychosine resurgence, demyelination, and neuroinflammatory cascades [41]. Psychosine exacerbates neurodegeneration by concurrently disrupting autophagy-lysosomal flux and UPS activity, driving cytoplasmic accumulation of cytotoxic aggregates of p62/ubiquitin and oligodendrocyte apoptosis [4]. In present study, aged AAV9-Twi mice lack detectable p62/ubiquitin aggregates in the brainstem and spinal cord and demonstrate resolution of neuroinflammation comparable to aged WT controls. This preservation of proteostasis and myelin integrity, evident in ultrastructurally normal sciatic nerves and sustained levels of myelin proteolipid protein, is correlated with the global GALC activity of the CNS/PNS and normalization of psychosine. Our data establishes that the current AAV9 delivery approach, by ensuring extensive enzyme distribution, preemptively abrogates psychosine’s dual neuroinflammatory and proteotoxic effects, thereby interrupting the self-perpetuating cycle of oligodendrocyte loss and neuronal dysfunction. These findings underscore the critical necessity for spatially uniform, lifelong GALC expression to achieve durable therapeutic rescue in GLD.

This streamlined intracranial AAV9 gene therapy protocol achieves unprecedented efficacy in GLD without adjunctive therapy, HSCT, multi-dose and/or multi-route CNS administration, or high systemic dosing. Our protocol ensures efficient neuronal transduction and broad biodistribution in both CNS and PNS, leading to sustained global correction of metabolism, prevention of neuroinflammation, preservation of proteostasis and myelin integrity, restoration of motor function, and maximal survival to dat. Remarkably, treated mice achieve lifespans approaching those of WT controls, suggesting durable and potentially lifelong therapeutic benefit. Although late-stage behavioral assessments were based on a small cohort, the findings are reinforced by consistent motor performance, stable body weight, preserved histopathology, and video documentation of aged animals (Video S1), supporting the maintenance of meaningful neurological function. These outcomes redefine the therapeutic benchmark for GLD, demonstrating that a streamlined, intracranial region-specific AAV9 monotherapy can achieve comprehensive neurobiological correction with minimal invasiveness. Translationally, this protocol provides a clinically scalable framework for human intracerebral AAV delivery, enabling durable CNS correction while minimizing systemic exposure and procedural risk.

In present study, regional differences in vector genome copies likely reflect a combination of injection geometry, neuroanatomical connectivity, and tissue volume dilution. Proximal regions such as thalamus and cortex showed higher vector genome copies, consistent with direct parenchymal delivery and dense local transduction, whereas lower levels in spinal cord and sciatic nerve are in line with secondary distribution via long-range axonal transport and cross-correction rather than primary deposition. In human brains, amplified white matter compartmentalization and 1000-fold larger volumes will exacerbate gradients, necessitating dose escalation, trajectory planning, and possibly combination therapies for ensuring robust enzyme activity in distal and peripherally located target tissues.

From a safety perspective, AAV9-treated Twi mice maintained stable body weight, normal behavior, and intact locomotor activity without late-onset neurological decline throughout their >800-day lifespan, supporting a favorable long-term safety profile. Nonetheless, several considerations remain important for clinical translation. Off-target transduction and peripheral organ involvement were not fully assessed and will require comprehensive biodistribution and toxicology studies. Vector- and transgene-specific immune responses, such as pre-existing anti-AAV9 antibodies and potential T-cell activation, may limit redosing in humans and necessitate careful immune monitoring. Although supraphysiological GALC expression was well tolerated in mice, overexpression toxicity remains a theoretical risk, underscoring the need for dose optimization in clinical applications. Overall, this streamlined protocol minimizes major known risks while achieving durable CNS and PNS correction, but also highlights key parameters to be addressed in future translational development.

## 5. Conclusions

While prior multi-route strategies and combination therapies have demonstrated meaningful benefits in GLD, particularly in enhancing PNS correction and mitigating neuroinflammation, they also involve substantial procedural complexity and systemic vector exposure. Our findings position the current single-dose, region-specific AAV9 approach as a complementary alternative that achieves comparable or superior long-term efficacy with reduced treatment burden. Recognizing the strengths of previous approaches underscores the significance of achieving such robust CNS–PNS correction through a streamlined and minimally invasive protocol.

## Figures and Tables

**Table 1:** Comparison of AAV-based therapeutic strategies in Twitcher mice.

Year	AAV Serotype	Postnatal Day	Route(s) & Sites	Vector Dose	Combination Therapy	Median Survival (Days)	Max. Survival (Days)	Reference
2005	AAV1	P0-1	i.c. (2 sites) i.c.v.	3 × 10^10^ vg 3 × 10^10^ vg	None None	55 55	66 66	[28]
2005	AAV2 AAV5	P3 P3	i.c. (6 sites) i.c. (6 sites)	4.4 × 10^8^ vg 2.4 × 10^9^ vg	None None	47 52	57 62	[17]
2007	AAV5	P3	i.c. (6 sites)	2.4 × 10^9^ vg	+BMT	111	151	[16]
2011	AAV5	P3	i.c. (6 sites) + i.t.	1.6 × 10^10^ vg, i.c. 2 × 10^10^ vg, i.t.	+BMT	71 123	78 282	[31]
2011	AAV5	P3	i.c. (6 site)	2.4 × 10^9^ vg	None	63	66	[29]
2012	AAVrh10	P2	i.c. (1 sites) + i.c.v. + i.v. (P2) + i.v. (P7)	1.5 × 10^9^ vg, i.c. 3.25 × 10^9^ vg, i.c.v. 7.6 × 10^9^ vg, i.v. (P2) 7.6 × 10^9^ vg, i.v. (P7)	None	104	240	[32]
2015	AAV5	P3	i.c. (6 sites)	2.4 × 10^9^ vg	None	60	63	[30]
2016	AAV9 AAVrh10	P10-11 P10-11	i.t. i.t.	2 × 10^11^ vg 2 × 10^11^ vg	+BMT +BMT	79 57	135 (Est.) 105 (Est.)	[36]
2018	AAV9	P0–P1	i.c. (5 sites) + i.t. + i.v.	9 × 10^9^ vg, i.c. 8.25 × 10^10^ vg, i.t. 3.3 × 10^11^ vg, i.v.	None +BMT	263 284	484 675	[34]
2021	AAVrh10	P8-9	i.v. i.v. i.v. i.v.	4 × 10^13^ vg/kg 4 × 10^13^ vg/kg 1.6 × 10^14^ vg/kg 4 × 10^14^ vg/kg	None +BMT None None	72 351 180 280	365 (Est.) 750 235 (Est.) 430	[33]
2021	AAV9	P0	i.c. (6 sites) + i.t.	1.2 × 10^10^ vg, i.c. 1.5 × 10^10^ vg, i.t.	None BMT BMT + SRT	66.5 269 404	83 675 569	[35]
2025	AAV9-	P3	i.c. (4 sites)	1.2 × 10^12^ vg, i.c.	None	530	814	Current study

BMT, bone marrow transplantation; Est, estimated; i.c., intracranial; i.c.v., intracerebroventricular; i.t., intrathecal; i.v., intravenous; SRT, substrate reduction therapy; vg, virus genome.

## Data Availability

All data generated or analyzed during this study are included in this published article.

## Supplementary Materials

The following supporting information can be downloaded at: https://www.mdpi.com/article/10.3390/cells14241942/s1, Video S1: AAV9-Twi Motor Activity. Representative videos showing an untreated Twi mouse at P40, WT mice at P57 and P768, and AAV9-Twi mice at P63 and P789. AAV9-Twi mice exhibited body size and locomotor activity comparable to age-matched WT controls, whereas the untreated Twi mouse was markedly smaller and displayed severe motor impairment.
